# Preoperative Embolization of Brain Tumors Using a Provocative Test: Evaluating the Safety and Efficacy of Embolization of Test-Positive Vessels

**DOI:** 10.7759/cureus.72650

**Published:** 2024-10-29

**Authors:** Takao Hashimoto, Hirofumi Okada, Kyosuke Matsunaga, Yusuke Arai, Muneaki Kikuno, Hiroki Sakamoto, Michihiro Kohno

**Affiliations:** 1 Department of Neurosurgery, Tokyo Medical University, Tokyo, JPN

**Keywords:** brain tumor, embolization, embosphere, provocative test, vasa nervorum

## Abstract

Purpose: The embolization of vessels potentially involved in the vasa nervorum during brain tumor embolization is often a non-aggressive procedure. In this study, we aimed to investigate the safety and efficacy of embolization of vessels with positive provocative test results.

Methods: Embosphere (Merit Medical Systems, South Jordan, Utah, USA) was the embolization material of choice. A provocative test was performed using 30 mg of 1% lidocaine, with a positive result if neurological symptoms appeared. A size of 500-700 µm was used for positive results and 300-500 µm for negative results. Embolization was performed five to seven days before resection, and contrast-enhanced MRI was performed three to five days after embolization to assess the embolic status.

Results: Provocative tests were performed on 61 vessels from 55 patients. A total of 36 vessels (59.0%) were positive, and 25 vessels were negative. The petrosal branch of the middle meningeal artery and the neuro-meningeal branch of the ascending pharyngeal artery showed particularly high positivity rates (91.7% and 76.5%, respectively). Three patients (8.3%) out of the 36 who underwent embolization of the positive vessels had neurological symptoms after embolization; two had symptoms similar to those observed in the provocative test, and one had different symptoms. All symptoms were mild and transient. The postoperative contrast-enhanced MRI showed that the enhancing effect was attenuated in 18 of the 30 patients (60%) with provocative test-positive vessels.

Conclusion: Embolization of provocative test-positive vessels can be performed safely and effectively with a larger Embosphere (500-700 μm).

## Introduction

Preoperative embolization of brain tumors is useful in reducing blood loss during resection, softening the tumor, and shortening the operation time by occluding the nutrient vessels of blood-rich tumors [1,2]. It is especially indicated for skull base tumors that are deep, with a difficult-to-expand surgical field and difficult-to-treat nutrient vessels [3,4]. However, the vasa nervorum is often involved. If the vasa nervorum is occluded by embolization, there is a risk of neurological symptoms; therefore, embolization should be avoided, or embolization should be performed on the proximal side of the feeding vessels to preserve the vasa nervorum network. In this study, we investigated the safety and efficacy of embolization using Embosphere (Merit Medical Systems, South Jordan, Utah, USA) in vessels that were determined to have vasa nervorum involvement by performing a provocative test on the vessels.

## Materials and methods

We performed preoperative embolization of large or deep brain tumors with high tumor staining on cerebral angiography. Embolization was performed five to seven days before resection, and contrast-enhanced MRI was performed three to five days after embolization. The microcatheter was guided to the periphery so that it would not wedge into the feeding vessel, and 30 mg of 1% lidocaine was injected for the provocative test. The test was judged positive if cranial nerve (CN) symptoms appeared and negative if no CN symptoms appeared. Positive results indicated vasa nervorum involvement, wherein embolization of the vessel could cause symptoms similar to those observed in the provocative test. Embosphere was the first choice of embolization material; for positive vessels, a larger size (500-700 µm) was used to prevent straying into the vasa nervorum; for negative vessels, a size of 300-500 µm was used. Embosphere had a particle volume of 2 mL dispersed in saline and a total volume of 9 mL in a 20 mL syringe. This was used as the undiluted solution and was diluted with a half-diluted contrast medium. Inject 300-500 μm at 30× dilution and 500-700 μm at 60× dilution into the bloodstream, avoiding press-fitting. In principle, the proximal side of the vessel was occluded with a platinum coil to prevent reopening. However, if the test was negative but there was an anatomical risk of the Embosphere straying into the vasa nervorum, a 500-700 μm Embosphere was used.

Of the 198 patients who underwent preoperative embolization between January 2017 and December 2023, 55 patients (61 vessels) who underwent embolization with a provocative test of the external carotid artery and meningohypophyseal trunk (MHT) were included in the study. The safety and efficacy of embolization of the provocative test-positive vessels were evaluated by assessing the presence of new neurological findings and attenuation of the enhancing effect on contrast-enhanced MRI after embolization. A contrast effect regression of 20% or more was defined as an attenuation of the enhancing effect.

## Results

A total of 55 patients (19 males and 36 females; age range, 18-73 years (mean, 48.1) were included, comprising 37 with meningiomas (all skull base meningiomas), 16 with schwannomas (including 11 vestibular schwannomas), one with an endolymphatic cyst, and one with a hemangioblastoma) (Table 1).

**Table 1:** Patient characteristics and vessels that underwent the provocative test AMA, accessory meningeal artery; APhA, ascending pharyngeal artery; PVT, provocative test; IMA, internal maxillary artery; MHT, meningohypophyseal trunk; MMA, middle meningeal artery; OA, occipital artery

		PVT Positive	PVT Negative	PVT Positive Rate (%)
Total number of patients	55	-	-	-
Age (years)	18-73 (mean 48.1)	-	-	-
Sex (male/female)	19/36	-	-	-
Diagnosis				
Meningioma	37 (all skull base meningioma)	-	-	-
Schwannoma	16 (vestibular schwannoma 11)	-	-	-
Endolymphatic cyst	1	-	-	-
Hemangioblastoma	1	-	-	-
Target artery	61	36	25	59.0
MMA (petrosal br/other)	22 (12/10)	14 (11/3)	8 (1/7)	63.6 (91.7/30.0)
AMA	3	2	1	66.7
IMA	2	0	2	0
APhA (all neuro-meningeal br)	17	13	4	76.5
OA (mastoid br/stylomastoid br)	4 (3/1)	3 (2/1)	1 (1/0)	75.0 (66.7/100)
MHT	13	4	9	30.8

Provocative test results

The 61 vessels for which the provocative test was performed included 22 middle meningeal artery (MMA), three accessory meningeal artery (AMA), two internal maxillary artery (IMA), 17 ascending pharyngeal artery (APhA), four occipital artery (OA), and 13 MHT. A total of 36 vessels (59.0%) were positive, and 25 vessels were negative. A total of 63.6% (14/22), 91.7% (11/12), 66.7% (2/3), 0% (0/2), 76.5% (13/17), 75.0% (3/4), 66.7% (2/3), 100% (1/1), and 30.8% (4/13) tested positive for MMA, petrosal branch, AMA, IMA, APhA (all neuro-meningeal branch), OA, mastoid branch, stylomastoid branch, and MHT vessels, respectively. In the vessels where the provocative test was performed more than 10 times, the MMA petrosal and APhA neuro-meningeal branches had high positivity rates of 91.7% and 76.5%, respectively.

Neurological symptoms after embolization

Embosphere having a diameter of 500-700 µm was used in 35 vessels with positive provocative test results, coils alone were used in one vessel, Embosphere having a diameter of 500-700 µm was used in 17 of 25 vessels with negative results, and Embosphere having a diameter of 300-500 µm was used in eight vessels.

After embolization, neurologic symptoms thought to be ischemia of the vasa nervorum appeared in three patients (three of 36 vessels, 8.3%) (Table 2), including petroclival, jugular foramen, and tentorial meningiomas. The included vessels were AMA, APhA neuro-meningeal branch, and MHT. All of them were positive for the provocative test. Two patients had the same symptoms as those observed in the provocative test, and one patient had different symptoms. Symptoms appeared 3-12 hours after embolization and improved within a few days in two cases and within approximately six months in one case, with no permanent residual symptoms. The embolization of vessels that were negative for the provocative test did not result in neurological symptoms.

**Table 2:** Three cases of ischemic symptoms in the vasa nervorum after embolization (＋) indicates a decrease in the enhancement effect on contrast MRI; (ー) indicates no decrease. AMA, accessory meningeal artery; APhA, ascending pharyngeal artery; MHT, meningohypophyseal trunk; CN, cranial nerve; MRI, magnetic resonance imaging

Age	Sex	Diagnosis	Size (mm)	Target Artery	Provocative Test	Embolic Material	Symptoms After Embolization	Contrast MRI Attenuation of Enhancement
53	F	Petroclival meningioma	56	AMA	Disorders of CN V, VI, VII	Embosphere 500-700, coil	Mild CN V and VI disturbances appeared after 12 hours. Improvement in 6 days.	(＋)
46	F	Jugular foramen meningioma	27	APhA neuro-meningeal branch	Disorders of CN IX, X	Embosphere 500-700, coil	Mild CN IX, X disorder appeared 3 hours later. Improvement in approximately 6 months.	(＋)
51	F	Tentorial meningioma	40	MHT	CN VI disability, hoarseness	Coil	Mild CN IV failure beginning 12 hours later. Improvement in 3 days.	(ー)

MRI findings after embolization

Contrast-enhanced MRI after embolization was performed in 48 of 55 cases; a 20% or greater reduction in contrast efficacy was observed in 18 out of 30 (60%) embolization cases involving provocative test-positive vessels and in 10 out of 18 cases (55.6%) of embolization involving provocative test-negative vessels.

An illustrative case

**Figure 1:**
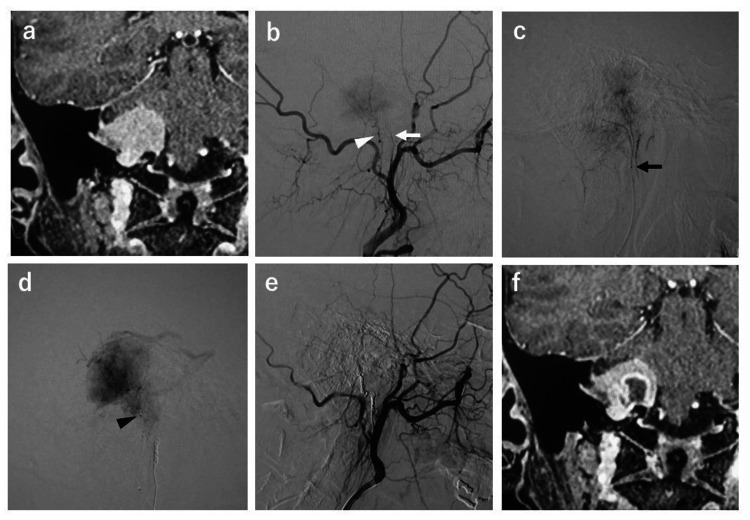
Contrast-enhanced MRI images of a 46-year-old female with right-sided hearing loss (a) Coronal view of contrast-enhanced MRI showing a 27-mm right jugular foramen meningioma with a uniform enhancing effect. (b) Lateral view of the right ECAG showing tumor staining from the rt-APhA neuro-meningeal branch (white arrow) and the right OA stylomastoid branch (white arrowhead). (c) Micro-contrast lateral view of the right APhA neuro-meningeal branch. Tumor staining is also observed. The catheter tip is indicated by the black arrow. (d) Micro-contrast lateral view of the right OA stylomastoid branch. Strong tumor staining is also observed. The catheter tip is indicated by the black arrowhead. (e) Lateral view of the right ECAG after occlusion of the rt-APhA neuro-meningeal branch and right OA stylomastoid branch with Embosphere 500–700 μm and a platinum coil. The tumor staining disappeared. (f) Coronal view of contrast-enhanced MRI performed five days after embolization showing a loss of contrast inside the tumor. ECAG, external carotid angiograms; APhA, ascending pharyngeal artery; OA, occipital artery

A 46-year-old female presented with right-sided hearing loss. Contrast-enhanced MRI showed a 27-mm-sized jugular foramen meningioma with a uniform enhancement effect (Figure 1). Rt external carotid arteriography showed tumor staining from the rt-APhA neuro-meningeal and right OA stylomastoid branches. A microcatheter was inserted into the right APhA neuro-meningeal branch and guided to the right APhA neuro-meningeal branch, where microangiography revealed tumor staining. A total of 30 mg of 1% lidocaine was used for the provocative test, and CNs IX and X were affected. After waiting for the improvement of symptoms, embolization was performed with an Embosphere of 500­-700 μm diameter, and the anterior part was occluded with a platinum coil to prevent recanalization. Next, a microcatheter was guided to the right OA stylomastoid branch, and microangiography revealed strong tumor brain staining. An Embosphere of 500-700 μm diameter was used for embolization without performing a provocative test because there was an anatomical risk of straying into the vasa nervorum. Subsequently, the front part was occluded with a platinum coil, and the tumor staining disappeared. Three hours after the end of embolization, mild CNs IX and X lesions, which previously appeared in a provocative test of the APhA neuro-meningeal branch, appeared. Symptoms improved over the course of six months. In addition, contrast-enhanced MRI performed five days after embolization showed a loss of contrast inside the tumor, indicating good embolization.

## Discussion

While previous studies [5-7] reported that the provocative test is useful in preoperative tumor embolization, it is often not performed because it is unreliable; false-positive and false-negative results may occur due to differences in the reach of the drug depending on the microcatheter position, injection speed, and reagent pressure. The injection rate and pressure of the drug are particularly important, as pressurized injection can lead to false positives due to backflow into another vessel. In this study, the reagent was injected at the same injection rate and pressure as during embolization whenever possible to avoid overpressurization and improve the reliability of the results.

Vasa nervorum

The vasa nervorum, which supplies blood to peripheral nerves, has long been described as a blood supply for CNs, with branches originating from the arteries near the CNs, crossing the epineurium and branching off within the perineurium to nourish the nerves [8].

The typical vasa nervorum includes the MMA petrosal branch to CNs VII and VIII, AMA to CNs V3, Vm, IMA artery of the foramen rotundum to CNs V2, APhA neuro-meningeal branch to CNs IX, X, XI, and XII, APhA inferior tympanic branch to CNs VII, OA mastoid branch to CNs VII, posterior auricular artery (PAA) stylomastoid branch to CNs VII, MHT tentorial artery to CNs III and IV, MHT dorsal meningeal artery to CNs IV, ILT to CNs III, IV, V1, and VI [9-14]. The facial nerves are supplied in the geniculate ganglion, and the lower CNs are primarily supplied in the foraminal portion [9].

There are no reports on the positive rate of provocative tests for these vessels. In this series, the MMA petrosal branch and APhA neuro-meningeal branch had high rates of 91.7% and 76.5%, respectively.

Provocative test-positive vessel embolization

Embolization of vasa nervorum vessels with a positive provocative test is not performed; however, proximal occlusion with a platinum coil is safer but less effective. In general embolization, CN palsy that appears with particle embolization is temporary and may recover after steroid therapy and with the development of collateral branches. In contrast, embolization with particles smaller than 80 μm or with liquid material often results in permanent cranial paralysis [9]. Particularly in skull base tumors, the vasa nervorum is frequently involved, and the use of embolic particles smaller than 150 μm should be avoided because of the increased risk of complications such as CN palsy [1,15]. Particles >300 μm and 300-500 μm are recommended for PVA (polyvinyl alcohol) and Embosphere, respectively [4,16].

In the present study, we used Embosphere 500-700 μm with a larger particle size because we judged that a positive result in the provocative test indicates the involvement of the vasa nervorum, which carries a very high risk of CN palsy. Of the 36 vessels that were positive in the provocative test, two showed CN palsy. One vessel had symptoms that were different from those observed in the provocative test; however, anatomically, it was thought to be an occlusion of the vasa nervorum. All these symptoms improved without becoming permanent. The use of a large Embosphere (500-700 μm) may have resulted in an improvement due to residual collateral blood circulation to the nerves even after the development of CN palsy. CN palsy did not occur in the 25 vessels for which the provocative test was negative, suggesting that the provocative test is a useful tool for predicting the risk of CN palsy associated with vasa nervorum occlusion and for determining the size of the Embosphere.

Approximately 3.7%-5.6% [1,17,18] of embolization complications have been reported. Rosen et al. [19] reported permanent complications in 9% of 167 embolization cases for skull base meningiomas. Considering that embolization was performed on a vessel at risk with a positive provocative test and that all symptoms were mild and transient, the 8.3% risk of cerebral palsy in this series was acceptable, and embolization may be considered if these vessels are difficult to treat at the time of removal.

In cases with good embolization, contrast-enhanced MRI after embolization has been reported to attenuate the enhancing effect [20-24]; if the regression of the contrast effect is stronger, intraoperative blood loss is reduced [20,24]. In this series, contrast-enhanced MRI was used to determine the embolic effect. Although simple comparisons cannot be made because of the number of nutrient vessels involved, embolization of a provocative test-positive vessel using larger 500-700 μm Embosphere particles and a platinum coil resulted in a weakened enhancement effect in 60% of the patients with post-embolization contrast-enhanced MRI. This was comparable to the 55.6% of embolization of provocative test-negative vessels using more 300-500 μm Embosphere particles, suggesting that good embolization was achieved. The Embosphere has a high peripheral reach [25], and even with particle sizes as large as 500-700 μm, effective embolization is possible if the blood flow into the tumor is high. However, because the vasa nervorum has weak blood flow and a small vessel diameter, it is thought that the large Embosphere does not reach it. Hence, the tumor can be safely embolized. Determining the size of the Embosphere based on the results of the provocative test may improve the safety of embolization procedures.

The limitations of this study include the small number of cases, the presence of a certain percentage of false positives and false negatives in the provocation test, and the fact that embolization of vessels other than the provocative test-positive vessels was also performed; therefore, the extent to which embolization of the provocative test-positive vessels increased the embolic effect was not analyzed. In the future, it is necessary to examine more embolization cases with provocative test-positive vessels only.

## Conclusions

The incidence of neurological symptoms in the embolization of provoked test-positive vessels with larger Embospheres (500-700 μm) was acceptable. Symptoms, if any, were mild and transient. Therefore, this procedure was considered safe and effective.
